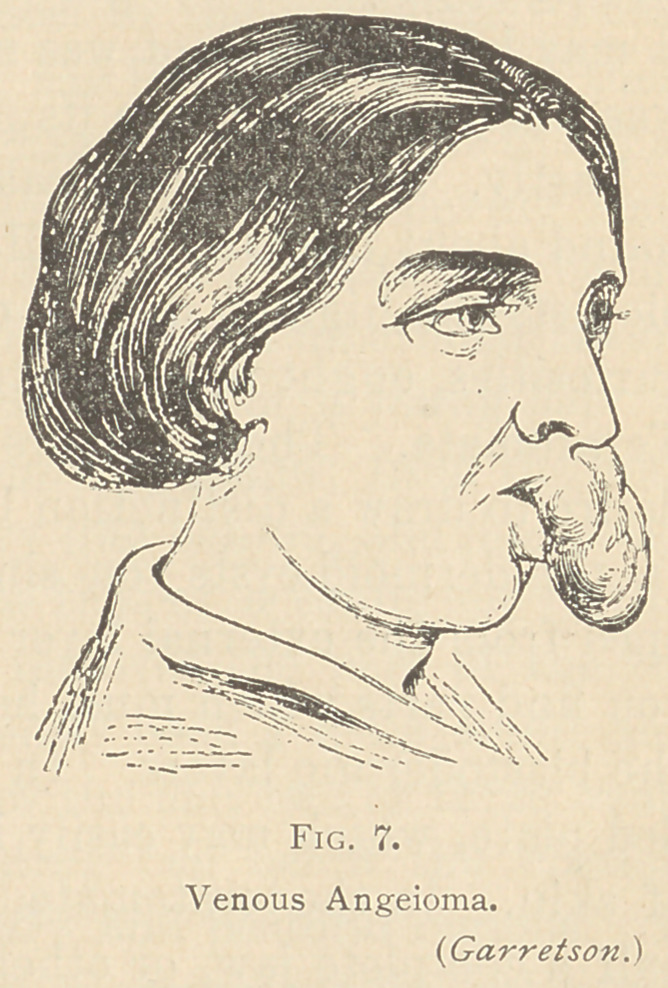# An Address on Congenital Deformities of the Mouth and Face

**Published:** 1888-06

**Authors:** Roswell Park

**Affiliations:** Professor of Surgery in the Medical Department of the University of Buffalo


### AN ADDRESS ON CONGENITAL DEFORMITIES OF THE MOUTH AND FACE.

BY ROSWELL PARK, A. M., M. D.

PROFESSOR OF SURGERY IN THE MEDICAL DEPARTMENT OF THE UNIVERSITY OF BUFFALO.

DELIVERED OCT. 25, 1887, BEFORE THE SEVENTH AND EIGHTH DISTRICT DENTAL SOCIETIES OF THE STATE OF NEW YORK, AND SPECIALLY REPORTED FOR THE INDEPENDENT PRACTITIONER.

*Mr. President and Gentlemen:—*

I fully appreciate the honor that your committee conferred upon me when they invited me to address you to-night. In casting about for a subject, it occurred to me that the time could not be more profitably spent than in a consideration of Congenital Deformities of the Mouth and Face. Some of these are common, others you may never see. If you are not already familiar with the method of operating on those that you do see, it is well that you should know how they may be treated; and of those that you seldom or never see, there are many that are very peculiar and interesting.

Fig. 1.

Head of Human Embryos; A, at end of third week; B, at end of fifth.

A 4, and B 6, Superior Max. processes.

A 6, and B 7. Inferior do., the latter now united in the middle line.

3. Naso-frontal process.

X. The tongue.

8. The first pharyngeal cleft.

(*From Ecker.*)

What I am about to say to you on this subject has so much to do with the embryology and early formation of these parts, that I propose for a moment to review the formation of the face. (See fig. 1, A and B.) I want to call your attention to the downward projection, which is the so-called naso-frontal process, and to these comparatively smaller points (A 4 and B 6), which are the superior maxillary processes from which are developed, a little later, the superior maxillæ. These grow from their present lateral position and unite with the naso-frontal process, which is necessary to the formation of the upper jaw. The second projections are the so-called inferior maxillary processes, which also meet in the middle line and form the lower jaw. This, the second post-oral process, is interesting to us, for from this is developed the upper part of the hyoid bone.

Let us turn to the same ovum three weeks later, at the end of the sixth week. (Fig. 1 B.) All the anterior parts of the skull, that is, the portion in conjunction with the brain, the eyes and their sacs, are developed more, and the naso-frontal process now has quite an identity of its own. The superior maxillary process has extended further toward the middle line from its rudimentary position, as shown in the first diagram. On the side is the so-called lateral mass, that comes down from the frontal and ethmoid bones and unites with the naso-frontal process to form the nose, and with the superior maxillary process to help form the upper jaw and the lateral walls of the nose. In the middle is the rudimentary tongue, which has a size disproportionate to the rest of the upper part of the embryo. The completed lower jaw, sometimes called the mandibular arch, is now plainly seen (B 7).

At the third week it has advanced pretty close to the middle line, but has not yet united across that line, while at the sixth week it has united. If you bear in mind these facts, it will very clearly explain those more common defects, such as hare-lip and cleft-palate, and will also account for these exceedingly rare and excessively horrible deformities which are sometimes seen across the whole face. Fortunately, infants having such deformities usually die soon after birth; sometimes, however, they grow up.

CLEFTS.

The most common clefts are cleft-palate and hare-lip. We cannot well understand the formation of cleft-palate without bearing in mind the development of the part. Fig. 2 is a representation of double cleft-palate, and the mass which projects is the lower aspect of the septum of the nose, carrying with it also the so-called intermaxillary bone. This bone is developed in some of the lower animals, and in some of them never completely unites—that is, it never fuses with the adjoining superior maxillary bone. A line of suture always exists between these bones, such as is found between the various bones of the cranium, but in the human being this suture can be found only before the twenty-fifth week, after which it disappears. But occasionally this line of union between the intermaxillary bone—or, as it is called by some, the incisive bone—and the superior maxillary does not take place, and then we have a fissure which may be either unilateral or bilateral, and in that case we have a single or double cleft-palate. This intermaxillary bone is a result of the fusion of the naso-frontal and the superior maxillary processes. The main part of it is developed downward from the naso-frontal process, and when it fails to unite with the superior maxillary process, we have the cleft.

FIG. 2.

Projecting Intermaxillary Bone with Double Cleft.

(*Garretson.*)

Of these forms of cleft-palate there may be several varieties :—

(1.) The double cleft, which extends backward on each side of the middle line.

(2.) A common cleft running through the hard and the soft palate, in which case the uvula is generally thrown to one side and may not be recognizable.

(3.) A single lateral cleft, extending entirely through.

(4.) A cleft which does not involve the alveolar process or gum.

(5.) One in which the cleft extends only part way through the hard palate, coming up within say three-quarters of an inch of the alveolar border.

(6.) A small cleft of the uvula alone. This is the mildest of all, and is remedied by the simplest possible surgical procedure.

The conditions of which I propose to speak to you to-night are amenable to surgery only, and cannot be cured in any other way, though you may palliate them with apparatus which you well know how to devise. When the intermaxillary bone projects very far forward, as in fig. 3, it makes a doubly disgusting deformity. Here is a projection, apparently from the nose; that is, the growth is attached to the septum, and is almost on a level with the tip of the nose. It is hard enough to close the fissure at best, but when the case is complicated with this unwelcome mass, one hardly knows what to do. There are three ways of getting at such a projection as this; one is to force it back and try to maintain it there by sutures. In mild cases this may be done. The second way is something like this. Virtually, we have the septum of the nose carrying the intermaxillary bones forward; with cutting forceps a V-shaped piece is taken completely out, then seizing this mass firmly, it may be crowded back to its place and so held that it will adhere for life, if there be sufficient vascular supply; otherwise it will gangrene and drop off. The next thing to do, the last resort, is entirely to excise it and bring the parts together without reference to it.

These cases of cleft palate are too often complicated with hare-lip, and the question arises—What shall be done for the child; when shall we operate, and how much shall we do? Let me say this to you; when a case is complicated with hare-lip, attend to that first. It is astonishing how much can be done by gentle prolonged pressure. If you do nothing else than unite that labial fissure, you will then get the pressure of the lips. For one moment or one hour, that amounts to very little, but when it is kept up for month after month and year after year, it does a great deal; not in bringing the sides of the jaw together, so much as in preventing the face bones from separating as they grow to adult size, so that it is always worth while to operate on hare-lip early. How early? I have operated within two or three weeks of birth, and I know one surgeon who claims to have operated on a child on the sixth day from birth. There is not quite such hurry as that, but I would advise you to operate within two or three months, unless the child is sickly and not likely to live. If it is in the hottest weather and there bids fair to be marasmic trouble, and the child is already delicate, you may say that it will not pay to torture the infant, which will die at all events.

One needs so much room for staphylorraphy that it is usual to wait till the child is five or six years old, operating immediately on the hare-lip, and feeling quite sure that the fissure will not widen any if it does not actually approximate. Quite recently, however, the Germans are advocating early operation for cleft-palate.

Fig. 3.

Hare Lip. Fistulæ of Lower Lip.

(*From Rose.*)

You may have simple hare-lip, uncomplicated by any fissure in the palate or alveolar border at all. Practically, if the cleft exists, it is almost always on one side or the other of the median line; it is very rare in the middle line. You may have single or double hare-lip. Garretson's Oral Surgery, which I suppose you all have, gives full descriptions of methods, and it is unnecessary for me to recapitulate them. I do not, however, always use hare-lip pins, and, for that matter, my method of operating differs a little from the classical method, but not so much so as to make it important to say anything about it. It is always advisable in operating on hare-lip to pare your fissures and get fresh edges, but not to cast away any integument until you find that you cannot utilize it. When the two edges are brought together, you will, perhaps, find that you greatly need the little part of skin, and if it had been cut completely off it would have been a mistake, for it can often be used to complete the continuity of the upper lip.

The question is sometimes raised as to the benefit of operations on cleft-palate with respect to articulation and deglutition. Many a patient is willing to go through life with a cleft-palate, because it does not show unless he or she opens the mouth, provided they cannot be assured that the speech is to be improved. You all know the excessively disagreeable nasal twang which this fissure causes, and it is a very important thing to know whether we can promise a person that he can speak better. Such patients will put up with the disgusting dropping of mucus into the mouth, and the discomfort of food passing up into the nose, if they cannot be assured that their articulation will be bettered by an operation. A child six years of age will not have learned to talk very much by that time, and if you operate on it early, the probability is that you will effect a great improvement by the time it is ten or twelve; but if the patient waits till he is twenty or thirty, and then undergoes an operation with the hope of improving his speech, both patient and operator will probably be disappointed; so it is always enough for me to operate in such cases for the sake of correcting the disgusting dropping of mucus and the inconvenience of deglutition, and not on account of the improvement of speech.

ATRESIÆ.

Such a thing as absolute closure of the nostrils has been met with, but it is rare. One or two cases are on record in which the nose has been occluded by bony union, so that it has been necessary to go through the obstruction with a bone drill. Membranous atresia is not so rare, though still uncommon. Sometimes the membrane is thick and tough, sometimes the merest film. Another deformity is that the nostrils are congenitally small. One sees complete closure of the nostrils, anteriorly, as the result of disease, as from cicatricial contraction, the result of syphilitic ulceration, and I have seen similar cases from diphtheritic lesions, but such cases are quite uncommon, and this congenital narrowing is still rarer.

Three cases of congenital narrowing of the posterior nares have been reported in Ziemssen's Cyclopedia. Closure of the posterior nares by diphtheritic or syphilitic processes is by no means uncommon. I have a woman at the hospital to-day, under my care, whose pharynx is almost shut off from the mouth by superficial ulceration, which has drawn down the palate so that there is almost no communication between the mouth and the pharynx proper. I have seen a similar case in a boy suffering with hereditary syphilis.

FISSURES AND DEFICIENCIES OF THE NOSE AND MOUTH.

Complete absence of the nose has been noticed in an infant at birth, and once in a while these patients have grown up and the absence of the nose has had to be atoned for by some mechanism, or by a plastic operation. When the nose is entirely absent, it is better to make some artificial contrivance rather than to perform the plastic operation.

Operations for fissures on one side of the nose and mouth have been made. (See fig. 4.) I am aware that these pictures are not lovely to look at, but they are after nature, and instructive, I hope.

FIG. 4.

Lateral Fissure of the Nose.

(*After Broca*)

So, too, lateral failure has been noted on one side of the face. Fig. 5 is a fissure extending from the mouth laterally, and it corresponds to a failure of closure between the superior and the inferior maxillary arches and the soft parts which cover them. It is very easy to explain if you refer back to the embryological development of these parts, but unless you do, it is a miracle.

The worst case of which I have any knowledge was that of a child which fortunately died when it was three days old. (See fig. 6.) This case presents a variety of fissures. There is almost complete absence of the palate. The tongue is rudimentary, and strange to say there is a bridge extending from the ordinary skin of the cheek up toward the superior maxillary bone, which there is no explaining, so we must call it an accident. But the whole thing, disgusting and unpleasant as it is, may be explained, with the exception mentioned, by reference to the embryology of the face and head. There are such things as double lateral fissures, occurring on both sides. The natural result of such deformity is to extend the mouth and make it spread, literally, across the face. That is one form of the deformity known as macro-stoma, which simply means large mouth. On the other hand we have the condition known as micro-stoma, which is a congenitally small mouth.

Median fissures of the face, affecting the upper jaw, are comparatively common; those of the lower jaw are uncommon; only three cases of median fissure of the lower jaw, tongue and hyoid bone, have been met with. Such defects in milder form are known, as, for example, when there is a false joint in the middle of the lower jaw, or no union at all, or splitting of the tongue, which, as you know, is the natural condition for serpents and some other reptiles. There are one or two cases on record in which the tongue has been divided into three parts instead of into halves.

Fig. 5.

Oblique Fissure of the Face.

(*From Pelvet.*)

Taking up for a moment atresia of these parts, there are cases on record of complete fixation of the tongue to the floor of the mouth. Much more frequent are partial illustrations of the same in tongue-tie, in which the frænum is too short. Simple splitting of that is enough. Don't be frightened by the statement made in some books that there is an artery in the frænum, and that the patient may bleed to death. Such a thing might occur, but there is very little danger of it. But there is danger of doing too much in this operation, for in some patients, who have already a long tongue, there might occur what is called "swallowing the tongue." One might imagine this an impossibility, but the tongue may be so long that it will get down into the throat and choke the patient. Cases of persons who could really swallow their own tongues have been exhibited. There are also instances of extreme elasticity and flexibility of the tongue, with unusual length. There was one man who could stand perfectly erect and put the tip of his tongue down to his chest, although it did not, in the mouth, appear abnormally long. That is, in one sense of course, a deformity. In another case the jaws had grown together simply by an adhesion of the gums, and not by a bony anchylosis.

Another case of extreme embryological interest, which is, so far as I know, unexplained and very rare, is that represented in fig. 3. This cut I used a moment ago to illustrate the projection of the intermaxillary bones, as it does, but here are two marks representing

FIG. 6.

Head of a three days old child. Specimen in the Bonn Patholog. Inst. Museum.

*a.* Cicatrized fissure of face.

*b.* Enlarged buccal aperture.

*c.* Intermaxillary bone.

*d.* Alveolar process.

*e.* A bridge of integument from the angle of the mouth to the gum.

Described by Remacly. (*From Trendelenburg.*)

fistulæ of the lower lip. There is nothing corresponding to it in the lower animals, or in the embryo, and this is an exception to a law which is something like this—that no matter what the deformity in the human species, you will find its analogue, either in the embryological life of the human fœtus, or in the lower animals. But I know of no analogue of this. Sometimes the fistula leads down from one and a half to two centimeters, and is simply a blind opening. Sometimes there are two fistulæ which converge, and sometimes they diverge. They seem to have no function whatever, and lead nowhere. Such a condition is easily remedied; it is the embryological interest it has which makes me allude to it.

CONGENITAL DEFICIENCY IN THE SIZE OF THE LOWER JAW.

There are cases on record in which the lower jaw does not match the upper jaw at all. These are usually accompaniments of fissures of the cheek and lips and other abnormalities. Langenbeck reported a case in which he could pass his hand into the child's mouth and feel that the ascending ramus was entirely absent, and then he could pass his finger up in the proper direction and feel the glenoid cavity of the temporal bone, and assure himself that it was empty. These deficient lower jaws usually have too few teeth.

Complete absence of the tongue is sometimes noticed. The most remarkable case on record is that reported by a French surgeon, some time ago. The tongue is sometimes found to be too small, and this results from a failure of the original tubercle, which represented the tongue, to develop properly. Then there is the so-called micro-glossus, which is simply the Greek word for small tongue, and, on the other hand, you and I will occasionally see a child whose tongue is too large for its mouth. Some of these patients cannot bring the teeth together without great effort, and these cases are always accompanied by tooth-marks on the sides of the tongue. That may be a congenital deformity, or it may arise in connection with the kind of tumor which we call lymphangeioma. You might call it elephantiasis of the tongue, for it is to the tongue what elephantiasis is to the skin of the limbs. The forms of macro-glossus are amenable to treatment, but micro-glossus is not. It is not possible to expand the tongue, though you may make it smaller by cutting out a piece, or by causing it to contract by the application of the cautery.

There is another disease known as macro-cheilia, which means large lip. One occasionally sees an immense overhanging upper lip, or an underhanging lower lip. This may be a congenital trouble, or it may be acquired. The case represented in fig. 7 is one of macro-cheilia, due to a venous tumor in the lip. It is a condition similar to macro-glossus—a kind of elephantiasis of the lip. Of course, as much of that as may be necessary may be cut away. One sees these venous tumors of the lip, beginning as a "mother's mark" or "strawberry mark," so-called, and if attended to early, they may be perfectly cured, but if put off, they may grow very large.

This same form of venous tumor may occur in the inside of the mouth. I have recently had under treatment and, I think, cured, a lady who had something of this kind in the inside of the mouth, spreading on the ramus of the jaw and down to the fauces. The remedy in her case was electrolysis. I also cured a young man in this city, of a corresponding condition, only worse, where the lower lip, upper lip and cheek were covered with such a tumor. I employed in this case a mixture of electrolysis and ligation.

Fig. 7.

Venous Angeioma.

(*Garretson.*)

Of the other tumors which are congenital in origin, let me refer to the papilloma, or warty growth. I have recently removed part of the tongue from a child which, when born, had a tumor on the front of his tongue; when presented to me the tongue was protruding from the mouth like a fist. It could not eat, could not drink, and could hardly nurse. I have also seen a child, the interior of whose mouth was covered with at least two hundred of these small papillomata.

Then the form of cystic tumor which comes underneath the tongue, known as ranula, may, sometimes, be of congenital origin, though this is in many cases an illustration of the formation of a cyst by occlusion. There is one form in which the duct is perfectly patulous, but which consists of a dilatation of the gland structure proper. The best treatment is complete excision of the cyst.

The so-called dermoid tumors are growths of no small interest. They are cysts containing more or less fluid, but they contain also such things as teeth and hair, and irregularly developed fragments of bone, and even other parts which grow from the external layer of the blastoderm, and they may be found in the mouth as well as anywhere else. A very interesting case of cystic tumor was reported in the Independent Practitioner a few months ago, and that is the best illustration I have seen. (See Vol. VIII, page 295.)

There are dentigerous cysts connected with abnormally developed, or, usually, misplaced teeth. You will find a tumor projecting into the mouth or against the cheek, which is hard, yet which seems to fluctuate; the cyst is opened into and you find a little fluid and a tooth. It may be that the tooth started too far down and could not grow up into the light; or it may be that the tooth started in the right position, but grew down or laterally instead of upward, or it may be that the root was not properly developed, and so the tooth was not pushed out into its proper place.

Lastly, I desire to speak of a class of tumors which may be found about the jaw, as well as anywhere else, which offer to pathologists the highest degree of interest, which are rare, and yet, as curiosities, ought to be mentioned to you to-night. They are the Teratomata. These tumors are connected with monstrosities. I desire to draw a distinction between dermoid cysts and teratomata. In the dermoid cysts you simply find such structures as are developed from the external layer of the blastoderm; in the teratomata you find parts which must have developed from two, or all three of the blastodermic layers. In the dermoid cyst you may find a calcified plate, which may easily have come from calcification of a piece of skin. In the teratomata, however, if you find a complete jaw or half a complete jaw, or other parts of a fœtus, it is a different matter. A case was reported some time since of a child who presented a large tumor on the face. On its removal and dissection there were found more or less complete parts of, apparently, a twin child. That did not mean twin pregnancy—it was an example of these rare growths, the teratomata.

Meyer reported, not long ago, a case in which a tumor was found growing on the lower jaw and on the side of the neck, and on its removal a complete lower jaw was found within it. You cannot have a complete lower jaw in a dermoid cyst; it was, therefore, a teratomata.

I have hurried over these points as briefly as I could to-night, and while there is much else that I should like to say to you concerning these matters, I will simply confine myself to one further abnormality, which does not exactly concern the mouth, but which comes up in connection with what has already been said. I refer to the bronchial cysts. You see between each of these arches (see fig. 1) a dark line representing a cleft. The arches start separately from each other, and later are drawn together, while the interval between them should be entirely closed. These fissures, by the way, are called bronchial clefts or fissures, because they correspond to the bronchiæ or gills of fishes, being to the human fœtus what the gills are to fishes. When they fail to close in the proper manner, they almost always give rise to cystic tumors. Such tumors extend, and, to a certain extent, migrate. Thus it may happen that one may be found extending from near the ear almost to the shoulder. These cysts are rare, and it is only within the last few years that their pathology has been made out, but it has fallen to my lot to see at least two of these rare cases, and in each the tumor—having been shut off probably before the child was born, at any rate long before I saw either of them—extended down beneath the superficial fascia, from the ear to the chest. In one case, that of a young lady, it extended from the ear to below the mammary gland; from the middle line in front to the posterior axillary line behind, and fluctuated under the clavicle. The other case was that of a young boy in this city, from whom another surgeon had removed the part of the tumor above the clavicle, but had apparently been a little timid and had not removed the rest. When I saw the lad, two or three years after the operation, there was a fluctuating bag below the clavicle reaching far downwards in the axillary and pectoral regions. It took a great deal of careful dissection to remove this, but it was done and I have the specimen yet, as a complete unbroken sac.

I have, perhaps, presented some things which you may think do not primarily concern you, but I know that you, as members of the great medical profession, are anxious to know something of matters outside of your own legitimate field.

## Figures and Tables

**Fig. 1. f1:**
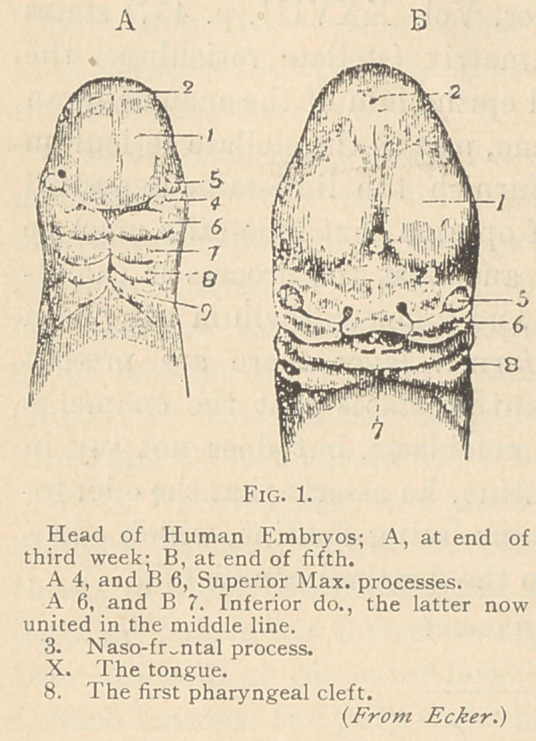


**Fig. 2. f2:**
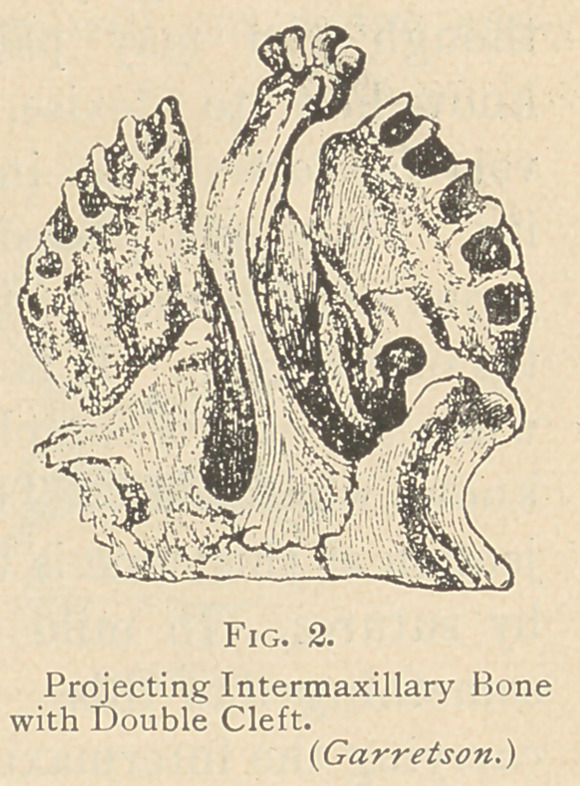


**Fig. 3. f3:**
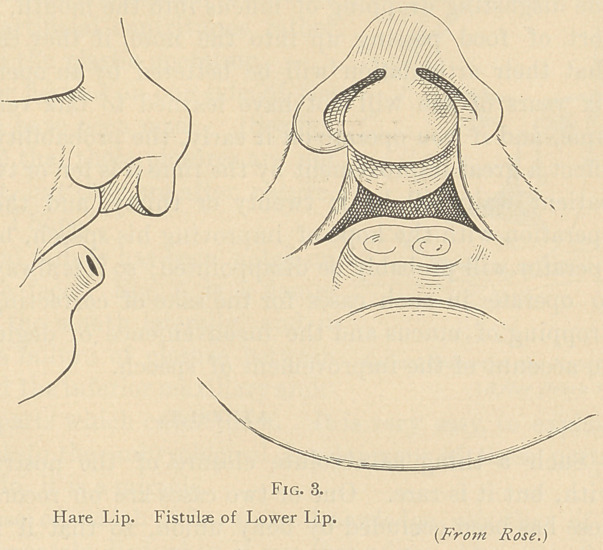


**Fig. 4. f4:**
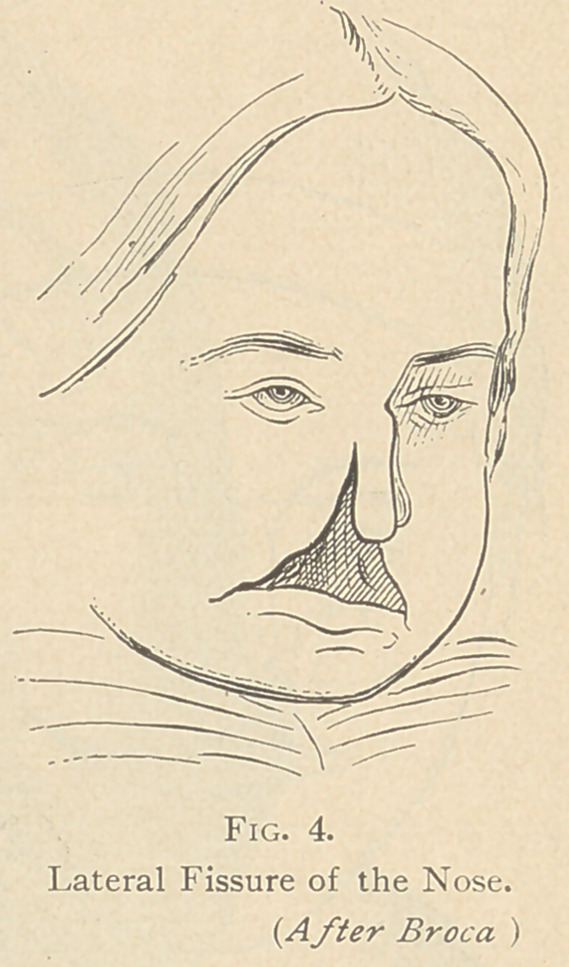


**Fig. 5. f5:**
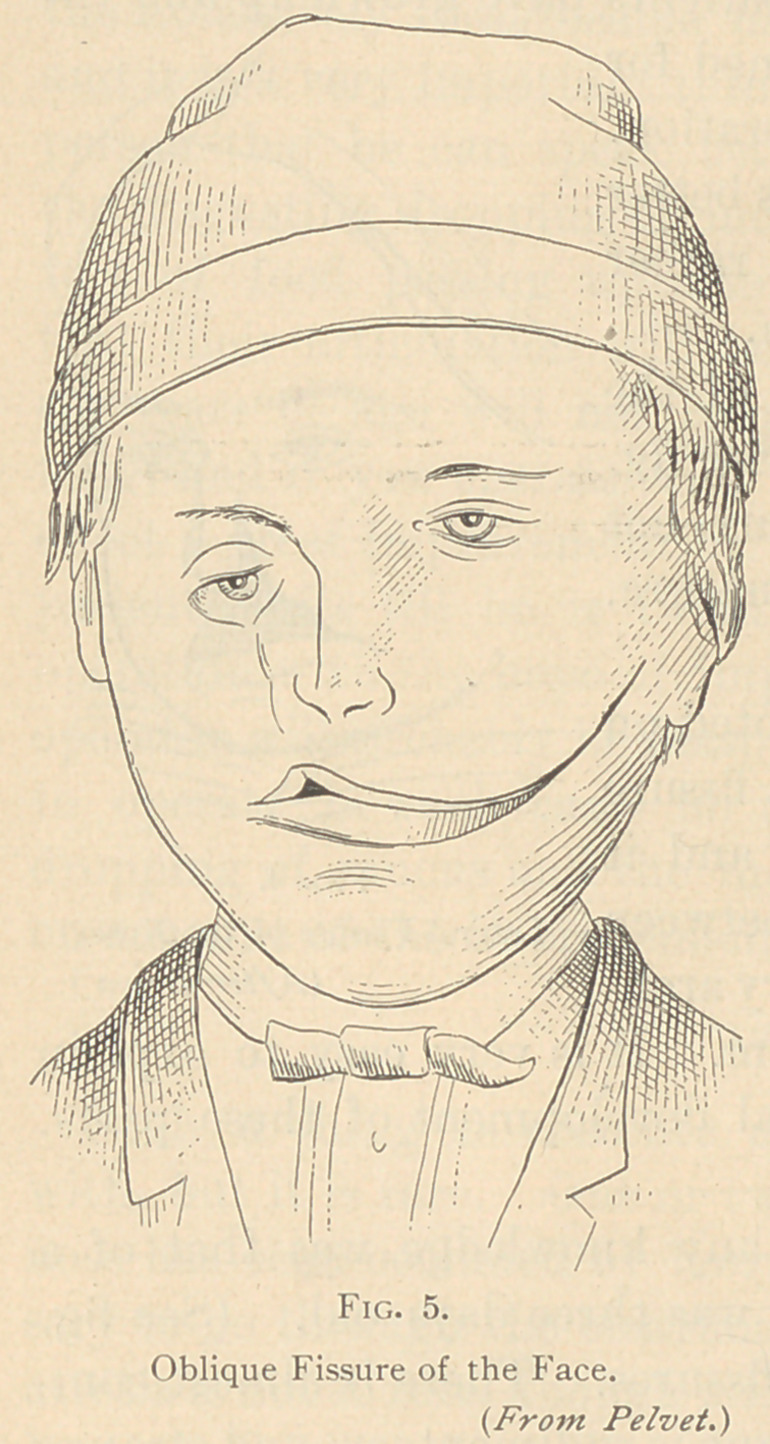


**Fig. 6. f6:**
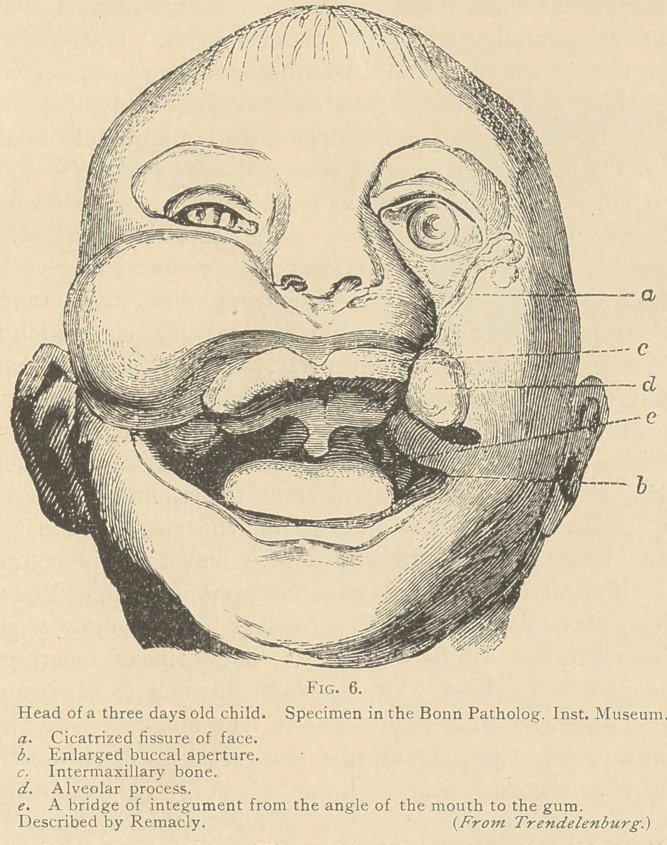


**Fig. 7. f7:**